# Incidence of renal cell carcinoma after solid organ transplantation: a systematic review and meta-analysis

**DOI:** 10.1186/s12894-023-01389-1

**Published:** 2024-01-06

**Authors:** Chang Xu, Hefeng Geng, Yannan Li, Fang Sun, Huiwei Sun, Yingshi Zhang, Qingchun Zhao

**Affiliations:** 1 Teaching Hospital of Shenyang Pharmaceutical University, General Hospital of Northern Theater Command, Shenyang, 100083 P.R. China; 2 Department of Clinical Pharmacy, Shenyang Pharmaceutical University, Shenyang, 110016 P.R. China; 3 Institute of Infectious Disease, Department of Infectious Disease, The Fifth Medical Center of Chinese PLA General Hospital, Beijing, 100039 P.R. China

**Keywords:** Incidence of renal cell carcinoma, Solid organ transplantation, Malignant incidence, Systematic review, Meta-analysis

## Abstract

**Background:**

The incidence rate of malignant tumors after solid organ transplantation is higher than the normal population. The aim of our study is to identify the risk of renal cell carcinoma (RCC) after liver, kidney, heart and lung transplantation, respectively, and suggest that transplant patients can be screened early for tumors to avoid risk.

**Methods:**

PubMed, Embase and the Cochrane Library from their inception until August 16,2023. Retrospective and cohort studies which focus on the statistical data of standardized incidence ratios (SIRs) of RCC after solid organ transplantation (SOT) more than one year have been included and extracted. The study was registered with PROSPERO, CRD4202022343633.

**Results:**

Sixteen original studies have been included for meta-analysis. Liver transplantation could increase the risk of RCC (SIR = 0.73, 95%CI: 0.53 to 0.93) with no heterogeneity(*P* = 0.594, *I*^2^ = 0.0%). And kidney transplantation could increase the risk of RCC(8.54, 6.68 to 10.40; 0.000,90.0%). Besides, heart and lung transplantation also could increase the risk of RCC(SIR = 0.73, 95%CI: 0.53 to 0.93; SIR = 1.61, 95%CI:0.50 to 2.71). Moreover, significance could also be found in most subgroups, especially the European group and retrospective study group. What’s more, after removing studies which have a greater impact on the overall outcome in RCC rate after kidney transplantation, heterogeneity did not solve and significant different was also observed in the European group (7.15, 5.49 to 8.81; 0.000, 78.6%).

**Conclusion:**

Liver, kidney, heart and lung transplantation patients have an increased risk of processing RCC compared to the general population and most subgroups, especially in geographic location of European subgroup, which suggested that patients should be screened frequently after transplantation.

**Supplementary Information:**

The online version contains supplementary material available at 10.1186/s12894-023-01389-1.

## Introduction

Although solid organ transplantation (SOT) has been the most useful alternative therapeutic strategy for solid-organ diseases nowadays [1, 2], which offers life-saving treatment for end-stage diseases considered terminal or those associated with a premonitory impairment in patients’ quality of life. Although immunosuppression therapy has also seen advantages with the expansion of immunosuppressive protocols to dampen the host immune response and improve short and long-term graft survival, which is accompanied by the risk of developing a postoperative malignant tumor [1, 3], and the incidence of malignant tumors after SOT is 2–5 times that of the normal population [4, 5]. Therefore, we need to conduct a combined analysis on the incidence of malignant tumors after important SOT.

Immunosuppressive therapy after transplantation reduces transplant recipients’ ability to control virus infections, thus exposing the recipients to increase risk of infection-associated cancers [6]. Moreover, the use of immunosuppressive agents to prevent allograft rejection increases the long-term risk of malignant tumor. Among the risks of solid tumors after transplantation, renal cell carcinoma (RCC) is the most common type. The cause may be driven primarily by kidney failure-related factors, with immunosuppression contributing to a lesser degree [7, 8]. So we determine to research the effect of important SOT on the incidence rate of RCC.

In this systematic review and meta-analysis, we determine to identify the risk of RCC after liver, kidney, heart and lung transplantation, respectively. And suggests transplant patients can be screened early for tumors to avoid risk. None of previous systematic review [9, 10] has provided a comprehensive overview with meta-analysis and meta-regression which to evaluate the transplant population most likely to progress to RCC.

## Materials and methods

This systematic review and meta-analysis followed systematic reviews and meta-analyses (PRISMA) guideline [11] and registered with International prospective register of systematic reviews(PROSPERO) website (NO. CRD4202022343633) [12].

### Search strategy and eligibility criteria

We meticulously searched electronic databases of PubMed (http://www.ncbi.nlm.nih.gov/pubmed), Embase (https://ersp.lib.whu.edu.cn/s/com/ovid/dc1/ovidsp/G.https/ovid-b/ovidweb.cgi) and the Cochrane Library (https://www.cochranelibrary.com/) from their inception until May 31, 2022; and update literature search was performed in August 16,2023. Search keywords were organ transplantation, incidence of renal cell carcinoma and their MeSH terms (see details in Table S1). Studies which focus on the statistical data of standardized incidence ratios (SIRs) of RCC after SOT more than one year have been included and extracted. Research type of either retrospective study or cohort study with no language restriction. Transplantation types include kidney, liver, heart and lung transplantation have been included, and no restrictions on nationality, region, time of transplants or follow-up time. The search process was performing by two independent researches (WY and LYN), and the controversy and disagreement have been resolved by the third experienced review(GHF or ZYS). Besides, the reference lists of relevant meta-analysis was also identified for potential eligible publications.

### Data collection and risk of bias

For each eligible study, the above pairs of researches extracted data independently using a standardized table. Baseline characteristic of first author, publication year, region, research type, research period, research design, number of transplant recipients, age, gender, follow-up period and incidence cases of RCC. For outcomes in this systematic review and meta-analysis, only SIR data with combined effect size model have been extracted. Moreover, subgroups analysis were grouping by transplant type (liver transplantation, kidney transplantation, heart transplantation, lung transplantation), geographic location (Europe, Asia, North America) and research type (retrospective study, cohort study). And the quality of eligible studies was assessed using the Newcastle-Ottawa Scale (NOS) score [13], and the score greater than 4 is acceptable. We also used the Grading of Recommendations Assessment, Development and Evaluation (GRADE) scales [14] to evaluate the quality of the outcomes from standard meta-analysis. All the above procedures were evaluated by two research separately, and disagreement have been discussion.

### Data synthesis and analysis

For combined effect size pairwise meta-analysis, in order to avoid homogeneity between included studies, random-effect models were used. And the heterogeneity among studies were assessed by *P*-value and *I*^2^ statistic, and *P*-values less than 0.05 or *I*^2^ more than 50% indicated heterogeneity in these outcome [15]. And, SIR data with their 95% credible intervals (95%CI) upper and lower limits have been extracted. Moreover, we take the natural logarithm of the above data and then merge them to avoid discrepancies in the data. Besides, the *P* value from meta-regression was used to determine whether the factor was the source of heterogeneity, *P* value less than 0.05 proved existences [16, 17]. Moreover, the Begg’s and Egger’s test were used to assess the publication bias for available comparisons, *P* value less than 0.05 means yes. We also used Galbraith plot to determine heterogeneity among included studies. All the aforementioned analyses were performed using StataSE version 15.1.

## Results

### Description of included studies

**Fig. 1:**
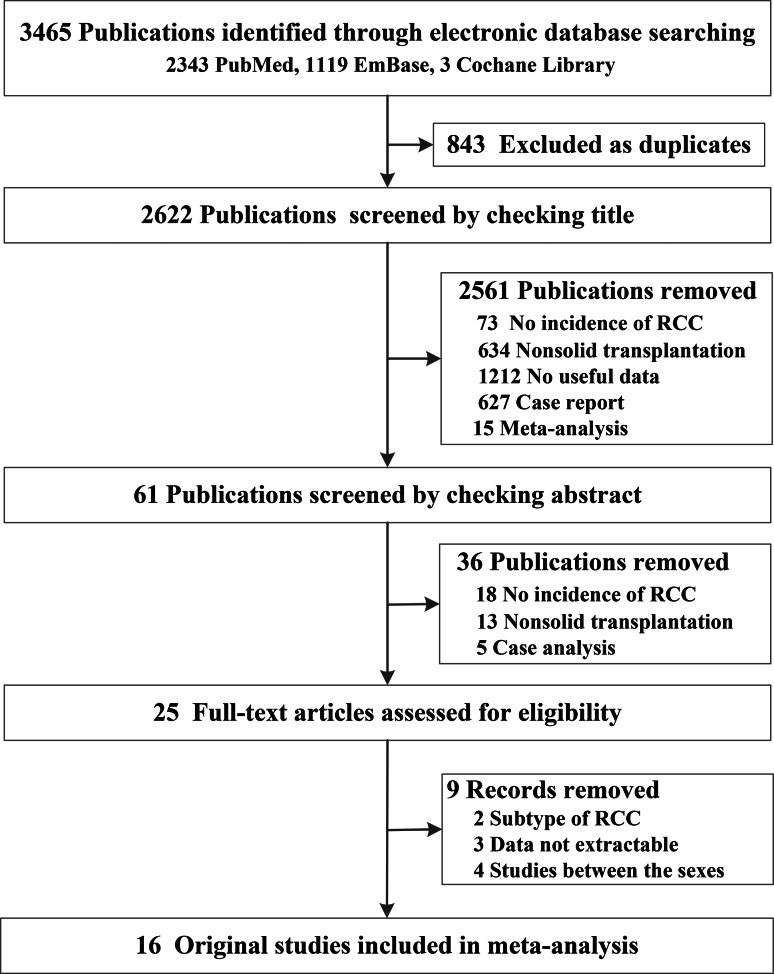
Flow chat of screening including original studies in meta-analysis

The electronic database search yielded 2622 unique publications, after screening by titles, 61 publications were left for checking. Then moving researches with no incidence of RCC, non-solid transplantation and case analysis, 25 publications were left for full-text checking. Later, studies of sub-type of RCC, no-extractable data available and researches between different sexes have been excluded. Ultimately, 16 original studies [18–33] have been included for meta-analysis, eleven of them reported incidence RCC data of liver transplant, kidney transplant (n = 11), heart transplant (n = 6) and lung transplant (n = 3)(Fig. 1). And seven of them were retrospective study, 9 of them were cohort study; the sample size was from 280 to 102,654; the baseline characteristic of incidence of RCC risk after transplantation were summarized in Table 1. The quality of all included studies was itemized in Table S2, they all scored 7–8 scales which were acceptable.

**Table 1:** Baseline characteristic of incidence of kidney cancer risk after transplantation

Study, year	Region	Research type	Research period	Research design	Number of transplant recipients	Age,gender(male%)	Follow-up period(year)	Incidence cases of kidney cancer
Friman TK,2022 [17]	Finland	Retrospective study	1987–2016	Liver transplantation	1078	49.8(36–58),49%	8.1(0-30.9)	7
				Kidney transplantation	4514	49.5(37–59),64%	9.6(0-30.9)	90
				Heart transplantation	599	50.3(37–57),75%	8.3(0-29.6)	11
				Lung transplantation	280	53.2(43–61),58%	4.5(0-25.8)	2
Leon-Rodriguez E,2020 [18]	Mexico	Retrospective study	1967–2015	Kidney transplantation	1404	36.5(16–70),58.2%	15(0–43)	8
Yeh CC,2020 [19]	Taiwan	Retrospective study	1997–2011	Liver transplantation	2127	45.5,71.6%	5.9(3.8)	
				Kidney transplantation	5038	44.4,52.9%	6.6(3.6)	
				Heart transplantation	687	46,79.8%	5.9(3.8)	
Lengwiler E,2019 [20]	Switzerland	Cohort study	2008–2014	Kidney transplantation	1557	53.9 (41.7–62.9),64.9	3.3(1.6-5)	--
O’Neill JP,2019 [21]	Ireland	Retrospective study	1994–2014	Liver transplantation	562	48.5(15.7–70.7),54.1%	--	2
				Kidney transplantation	2382	43.02(1.42–77.43),62.7%	--	11
				Heart transplantation	214	47.09(40.77–57.55),77.6%	--	4
Heo J,2017 [22]	South Korea	Retrospective study	1994–2014	Liver transplantation	2462	53,81.9%	12.4 M(0.8–53.0)	3
Kaneko J,2013 [23]	Japanese	Cohort study	1996–2012	Liver transplantation	360	49,53.3%	7.5 ± 3.4	2
Krynitz B,2013 [24]	Swedish	Cohort study	1970–2008	Liver transplantation	1221	49(36–57),57%	5(0–21)	2
				Kidney transplantation	7952	47(35–57),62%	8(0–38)	70
				Heart transplantation	1012	50(38–57),61%	5(0–23)	1
Piselli P,2013 [25]	Italy	Cohort study	1997–2009	Kidney transplantation	7217	-,64.2%	5.2(2.9–7.8)	36
Schrem H,2013 [26]	Germany	Retrospective study	1983–2010	Liver transplantation	2000	36.7(0–71), 53.3%	7.25	7
Cheung CY,2012 [27]	China Hongkang	Retrospective study	1972–2011	Kidney transplantation	4895	43.7 ± 12.6, 58.6%	4.8	26
Engels EA,2011 [28]	USA	Cohort study	1987–2008	Liver transplantation	37,888	47,60.9%		1
				Kidney transplantation	102,654			565
				Heart transplantation	17,981			85
				Lung transplantation	7013			8
Collett D,2010 [29]	UK	Cohort study	1980–2007	Liver transplantation	6846	− 0.52%		
				Kidney transplantation	25,104	− 0.62%		
				Heart transplantation	3609	− 0.80%		
				Lung transplantation	2058	− 0.53%		
Aberg F,2008 [30]	Finland	Cohort study	1982–2005	Liver transplantation	540	43 ± 18, 45%	6.3y(0–24)	2
Jiang Y,2008 [31]	Canada	Cohort study	1983–1998	Liver transplantation	2034	40–70,62%	42.2 ± 33.8 m	4
Villeneuve PJ,2007 [32]	Canada	Cohort study	1981–1998	Kidney transplantation	11,155	− 0.63.2%	-	71

### Incidence of renal cell carcinoma risk after transplantation

**Fig. 2:**
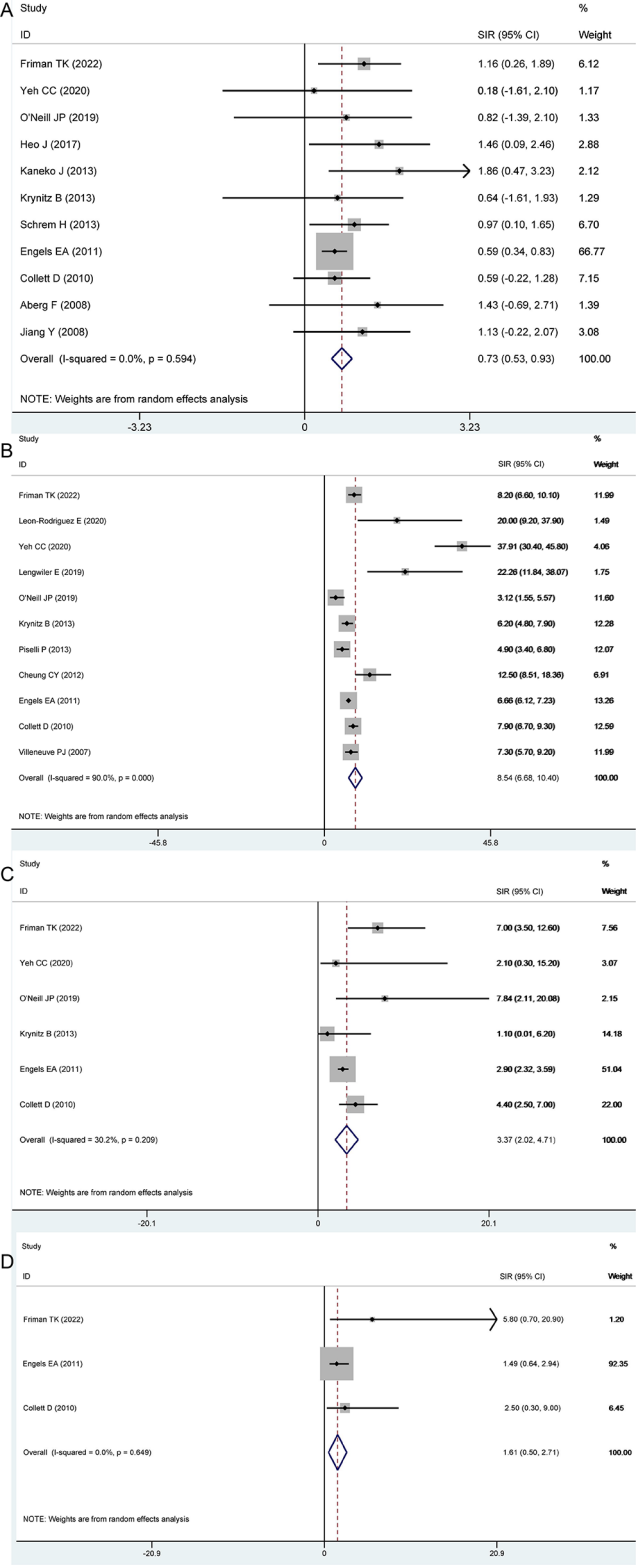
Forest funnel of renal cell carcinoma incidence after liver transplantation (**A**), kidney transplantation (**B**), heart transplantation (**C**), lung transplantation (**D**)

Table 2 and Fig. 2 summarized total data and subgroup analysis from original studies of the incidence of RCC in the liver, kidney, heart, and lung transplant patients. We can notice that in the first section, liver transplant could significant increase incident rate of RCC(SIR = 0.73, 95%CI: 0.53 to 0.93) with no heterogeneity (*P* = 0.594, *I*^2^ = 0.0%), and the publication bias could be tested from the Egger’s test (*P* = 0.036), and the GRADE assessment was low grade. Besides, significance could also be found in geographic locations of Europe (0.91, 0.50 to 1.32) and Asia (0.93, 0.27 to 1.59) subgroups, and research type of both retrospective study (1.89, 1.46 to 2.31) and cohort study (2.07, 0.88 to 3.26), with low heterogeneity, moderate grade and no publication bias.

**Table 2:** Incidence of renal cell carcinoma which subgroup analyzed by transplant type, region and research type

Transplant type	Study(patients)	SIR(95%CI)	Heterogeneity	Meta-regression	Publication bias	Grade
**Liver Transplantation**						
**Total**	11(57,118)	0.73 (0.53, 0.93)*	0.594, 0.0%		0.876, 0.036^¶^	Low
**Geographic location**						
Europe	6(12,247)	0.91(0.50, 1.32)*	0.906, 0.0%	0.945	0.851, 0.784	Moderate
Asia	4(42,837)	0.93(0.27,1.59)*	0.158,42.2%		0.497, 0.353	Moderate
North America	1(2034)	1.13(-0.01, 2.28)	-			-
**Research type**						
Retrospective study	6(46,117)	1.89(1.46, 2.31)*	0.767,0.0%	0.561	0.851, 0.218	Moderate
Cohort study	5(11,001)	2.07(0.88, 3.26)*	0.861,0.0%		0.624, 0.270	Moderate
**Kidney Transplantation**						
**Total**	11(173,872)	8.54(6.68, 10.40)*	0.000,90.0%^#^		0.881, 0.393	Low
**Geographic location**						
Europe	6(48,726)	6.43(4.55, 8.31)*	0.000,82.2%^#^	0.285	0.348, 0.805	Low
Asia	3(112,587)	18.50(4.33, 32.68)*	0.000,97.1%^#^		0.602, 0.508	Low
North America	2(12,559)	11.57(-0.19,23.33)	0.085,66.3%^#^		0.317,-	Very low
**Research type**						
Retrospective study	6(120,887)	11.85(7.82,15.89)*	0.000, 94.2%^#^	0.519	0.573, 0.468	Low
Cohort study	5(52,985)	6.83(5.23, 8.43)*	0.008, 71.0%^#^		0.624, 0.654	Low
**Heart Transplantation**						
**Total**	6(24,102)	3.37(2.02, 4.71)*	0.209, 30.2%		0.748, 0.276	Moderate
**Geographic location**						
Europe	4(5434)	4.17(1.49, 6.85)*	0.119, 48.7%	0.047^&^	1.000, 0.798	Moderate
Asia	2(18,668)	2.89(2.26, 3.53)*	0.834, 0.0%		0.317,-	Low
**Research type**						
Retrospective study	4(19,481)	3.93(1.58, 6.28)*	0.237, 29.2%	0.842	1.000, 0.389	Moderate
Cohort study	2(4621)	2.93(-0.29, 6.14)	0.091, 64.9%^#^		0.317,-	Low
**Lung Transplantation**						
**Total**	3(9351)	1.61 (0.50, 2.71)*	0.649, 0.0%		1.000, 0.312	Low

**Fig. 3:**
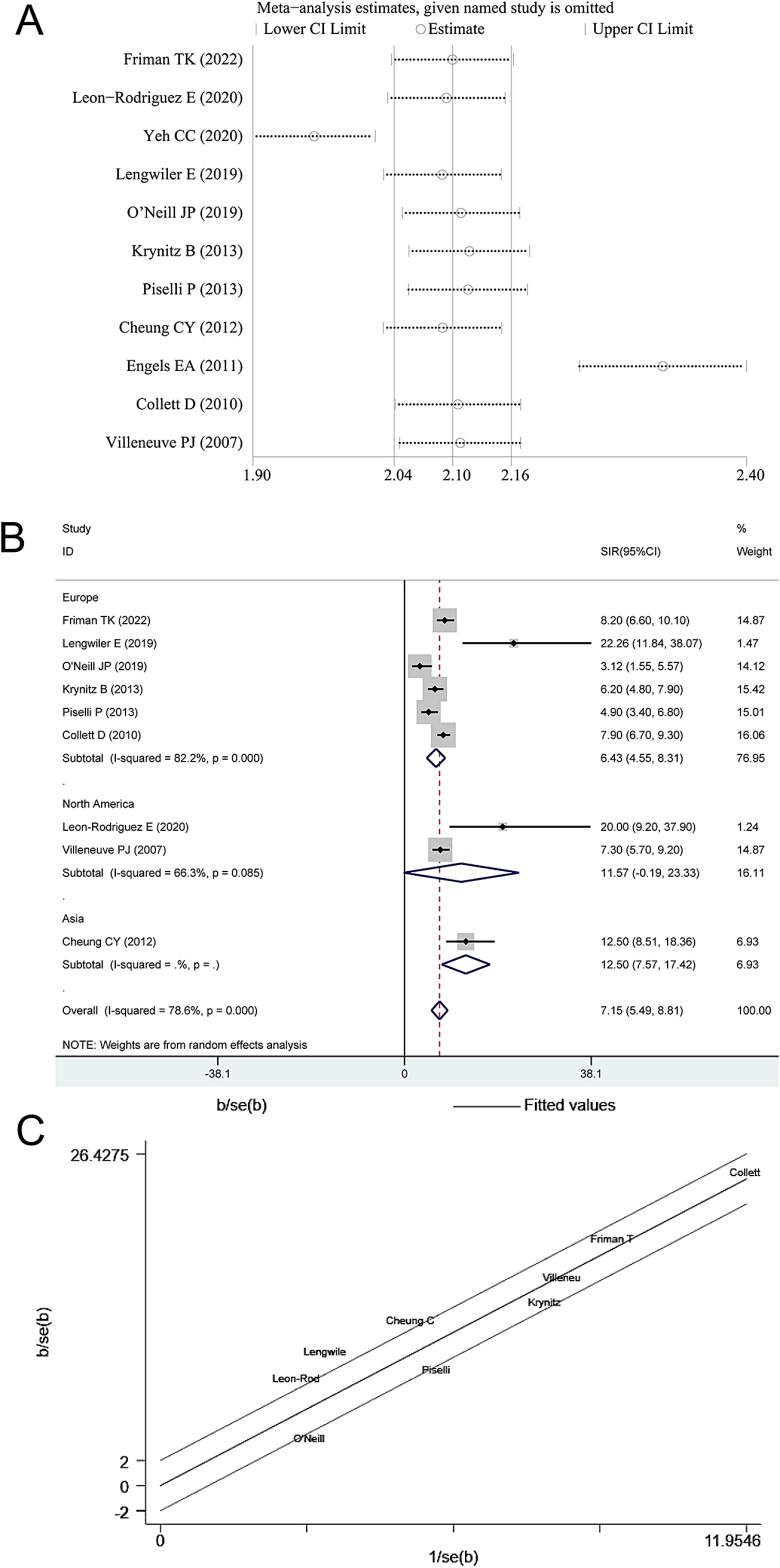
Sensitivity analysis of renal cell carcinoma (A), forest funnel (B) and Galbraith plots (C) of renal cell carcinoma incidence after kidney transplantation after remove Yeh CC and Engels EA

For SIR of RCC after kidney transplantation, significant different could be found in overall (8.54, 6.68 to 10.40), geographic location of Europe (6.43, 4.55 to 8.31) and Asia (18.50, 4.33 to 32.68) subgroups, retrospective study (11.85, 7.82 to 15.89) and cohort study (6.83, 5.23 to 8.43). Strangely, there was substantial heterogeneity within overall and all subgroups, and no source of heterogeneity was determined by meta-analysis. Therefore, sensitivity analysis was done for further analysis. From the outcome of sensitivity analysis, we found that the researches of Yeh CC(2020) and Engels EA(2011) may have a greater impact on the overall outcome (Fig. 3A). Subsequently, significance could be found in overall after the adjustment also with substantial heterogeneity (7.15, 5.49 to 8.81; 0.000, 78.6%; Fig. 3B), and geographic location of Europe (6.43, 4.55 to 8.31; 0.000, 82.2%). Galbraith plots were used for heterogeneity testing and we found that none of the adjusted data fell outside the confidence interval, suggesting less heterogeneity among included studies (Fig. 3C).

For incident risk of RCC after heart transplantation, significance could be found in overall(3.37, 2.02 to 4.71), geographic location of Europe (4.17, 1.49 to 6.85) and Asia (2.89, 2.26 to 3.53) subgroups and retrospective study (3.93, 1.58 to 6.28), with low heterogeneity and low-moderate grade. Besides, meta-regression detected different geographic regions may influence the existence of heterogeneity (*P* = 0.047). For the risk of RCC after lung transplantation, significant different with no heterogeneity could be found (1.61, 0.50 to 2.71, Table 2 and Fig. 2).

In summary, the incident of RCC risk could increase after solid organ transplantation, especially in the European subgroup.

## Discussion

This research followed standard PRISMA statement and registered with PROSPERO website. First, 16 original studies [18–33] were yield through the layers processing, 11of them provided SIR and their 95% data of liver transplantation, 11 of them provide data of kidney transplantation, 6 of them in heart transplantation and 3 of them with data of lung transplantation(Table 1; Fig. 1). Second, from pairwise meta-analysis, incident of RCC risk could increase from liver, kidney, heart and lung transplantation, and significance could find in most subgroups (Table 2; Fig. 2). Besides, meta-regression showed that grouping by geographic location maybe the source of heterogeneity. Third, sensitivity analysis determine that Yeh CC and Engels EA may had great influence on overall outcome of incident risk of RCC. After removing them, we could notice that SOT increase risk of RCC mainly in Europe group (Fig. 3).

From our research, although the incidence rate of RCC will increase after SOT, we also found that the incidence rate RCC after kidney transplantation is higher (8.54, 6.68 to 10.40) than that of incidence rate such as liver, heart and lung transplantation. Moreover, we do not know what causes the clinical heterogeneity among adjustment forest. Kidney transplant recipients have at least a 2–5 fold higher risk of developing or dying from malignant tumor than the general population. The increased risk of primary and recurrence cancer in transplant recipients is complex and attributed to oncogenic viruses, immunosuppression and altered T cell immunity [34]. The cause of heterogeneity may include different immunosuppressive agents, dose regimens, duration, etc. Immunosuppressant medication promotes impaired immunosurveillance, activation of oncogenic viruses, and have carcinogenic effects [34, 35]. Different studies have described an increased risk of RCC development in patients with chronic kidney disease (CKD). As a results of the chronic uremic state in patients and other former risk factors associated with both CKD and malignancy [36, 37]. Nowadays, risk factors for cancer-based on recipient characteristics known a pre-exist at the time of transplantation, such as sex differences that have not been well explored. And many medical disciplines have shown increasing awareness of how diseases manifest differently in men and women, trends in age-standardized cancer incidence rates differ by sex, being stable in men and increasing slightly among women during the most recent five years [38, 39].

The mechanism of incident of RCC, immunosuppressive agents could induce carcinogenesis by reducing immunosurveillance and mechanisms involved in the immunologic control of oncogenic viral infection or direct deoxyribonucleic acid (DNA) damage. However, the therapeutic effect of each drug on RCC development remains controversial because of the use of multi-agent therapy regimens. The increased risk of RCC may be mediated by the overall or cumulative exposure to immunosuppression more than by the agent itself [35, 36].

There are also some limitations among our study. First, only 16 publications have been included into meta-analysis, and for the incident risk of RCC after heart and lung transplantation, only 6 and 3 articles have been included, the results from meta-analysis may not be accurate enough. Second, the subgroup analysis only grouped by geographic location (Europe, Asia, North America) and research type (retrospective study, cohort study). As for the incidence rate of RCC, the baseline difference in follow-up time is huge, and some original studies do not report the follow-up time, so it is difficult for us to conduct subgroup analysis. Third, we only did sensitivity analysis for renal transplantation which with higher heterogeneity. After sensitivity analysis, the heterogeneity among the studies was still large, which may be the impact of clinical heterogeneity of incidence RCC rate.

In summary, the results of this systematic review and meta-analysis suggest that liver, kidney, heart and lung transplantation patients have an increased risk of processing RCC compared to the general population and most subgroups, especially in geographic location of European subgroup. We addressed the issue of post-transplant tumor incidence and performed geographic risk stratification analysis suggesting that European patients should pay more attention to early tumor screening after transplantation. Future patients-based retrospective and cohort study are required to explore the potential clinical risk factors associated with incident risk of RCC.

### Electronic supplementary material

Below is the link to the electronic supplementary material.

**Supplementary Material 1**: Table S1 Search strategies

**Supplementary Material 2**: Table S2 Risk of bias summary from NOS scale

**Supplementary Material 3**: PRISMA(2020) checklist

**Supplementary Material 4**: PRISMA Flow Diagram

**Supplementary Material 5**: CRD4202022343633

## Data Availability

All data generated or analysed during this study are included in this published article and its supplementary information files.
