# Supplementary material for: Incidence of renal cell carcinoma after solid organ transplantation: a systematic review and meta-analysis
Source: BMC Urol. 2024 Jan 6;24:11. doi: 10.1186/s12894-023-01389-1 (PMC10771683; doi:10.1186/s12894-023-01389-1)
Supplement: Supplementary file 2 — Supplementary Material 2: Table S2 Risk of bias summary from NOS scale [file 12894_2023_1389_MOESM2_ESM.doc]

**Table S2 Risk of bias summary from NOS scale.**

| **Publication(year)** | **Is the case definition adequate** | **Representativeness of the cases** | **Selection of Controls** | **Definition of Controls** | **Comparability of cases and controls (/2)** | **Ascertainment of exposure** | **Same method of ascertainment for cases and controls** | **Non-Response rate** | **Overall rating and TOTAL SCORE / 10** |
| --- | --- | --- | --- | --- | --- | --- | --- | --- | --- |
| Friman TK,2022[1] | 1 | 1 | 0 | 1 | 1 | 1 | 1 | 1 | 7 |
| Leon-Rodriguez E,2020[2] | 1 | 1 | 0 | 1 | 2 | 1 | 1 | 1 | 8 |
| Yeh CC,2020[3] | 1 | 1 | 0 | 1 | 2 | 1 | 1 | 1 | 8 |
| Lengwiler E,2019[4] | 1 | 1 | 0 | 1 | 1 | 1 | 1 | 1 | 7 |
| O'Neill JP,2019[5] | 1 | 1 | 0 | 1 | 2 | 1 | 1 | 1 | 8 |
| Heo J,2017[6] | 1 | 1 | 0 | 1 | 2 | 1 | 1 | 1 | 8 |
| Kaneko J,2013[7] | 1 | 1 | 0 | 1 | 2 | 1 | 1 | 1 | 8 |
| Krynitz B,2013[8] | 1 | 1 | 0 | 1 | 2 | 1 | 1 | 1 | 8 |
| Piselli P,2013[9] | 1 | 1 | 0 | 1 | 1 | 1 | 1 | 1 | 7 |
| Schrem H,2013[10] | 1 | 1 | 0 | 1 | 2 | 1 | 1 | 1 | 8 |
| Cheung CY,2012[11] | 1 | 1 | 0 | 1 | 2 | 1 | 1 | 1 | 8 |
| Engels EA,2011[12] | 1 | 1 | 0 | 1 | 1 | 1 | 1 | 1 | 7 |
| Collett D,2010[13] | 1 | 1 | 0 | 1 | 2 | 1 | 1 | 1 | 8 |
| Aberg F,2008[14] | 1 | 1 | 0 | 1 | 2 | 1 | 1 | 1 | 8 |
| Jiang Y,2008[15] | 1 | 1 | 0 | 1 | 2 | 1 | 1 | 1 | 8 |
| Villeneuve PJ,2007[16] | 1 | 1 | 0 | 1 | 2 | 1 | 1 | 1 | 8 |
