# Supplementary material for: Incidence of renal cell carcinoma after solid organ transplantation: a systematic review and meta-analysis
Source: BMC Urol. 2024 Jan 6;24:11. doi: 10.1186/s12894-023-01389-1 (PMC10771683; doi:10.1186/s12894-023-01389-1)
Supplement: Supplementary file 3 — Supplementary Material 3: PRISMA(2020) checklist [file 12894_2023_1389_MOESM3_ESM.docx]

**Identification of studies via databases and registers**

Records removed *before screening*:

Duplicate records removed (n = 843)

Records marked as ineligible by automation tools (n = 0)

Records removed for other reasons (n = 0)

Records identified from PubMed, EmBase, Cochrane:

Databases (n = 3465)

Registers (n =0 )

**Identification**

Records screened

(n = 2622)

Records excluded after checking titles

(n = 2561)

Reports sought for retrieval

(n =61 )

Reports not retrieved

(n =0 )

**Screening**

Reports assessed for eligibility

(n =61 )

Reports excluded:

No incidence of RCC (n =18 )

Nonsolid transplantation (n = 13)

Case analysis (n =5 )

Subtype of RCC(n=2)

Data not extractable(n=3)

Studies between the sexes(n=4)

Studies included in review

(n = 16)

Reports of included studies

(n =16 )

**Included**

*Consider, if feasible to do so, reporting the number of records identified from each database or register searched (rather than the total number across all databases/registers).

**If automation tools were used, indicate how many records were excluded by a human and how many were excluded by automation tools.

*From:*  Page MJ, McKenzie JE, Bossuyt PM, Boutron I, Hoffmann TC, Mulrow CD, et al. The PRISMA 2020 statement: an updated guideline for reporting systematic reviews. BMJ 2021;372:n71. doi: 10.1136/bmj.n71

For more information, visit: <http://www.prisma-statement.org/>
