# Supplementary material for: Incidence of renal cell carcinoma after solid organ transplantation: a systematic review and meta-analysis
Source: BMC Urol. 2024 Jan 6;24:11. doi: 10.1186/s12894-023-01389-1 (PMC10771683; doi:10.1186/s12894-023-01389-1)
Supplement: Supplementary file 5 — Supplementary Material 5: CRD4202022343633 [file 12894_2023_1389_MOESM5_ESM.doc]

**Table S1 Search strategies**

**Search strategies for PubMed**

**transplantation:** "transplantability"[All Fields] OR "transplantable"[All Fields] OR "transplantated"[All Fields] OR "transplantating"[All Fields] OR "transplantation"[MeSH Terms] OR "transplantation"[All Fields] OR "transplantations"[All Fields] OR "transplanted"[All Fields] OR "transplanting"[All Fields] OR "transplantation"[Subheading] OR "transplantation's"[All Fields] OR "transplanter"[All Fields] OR "transplanters"[All Fields] OR "transplantion"[All Fields] OR "transplants"[MeSH Terms] OR "transplants"[All Fields] OR "transplant"[All Fields]

**incidence:** "epidemiology"[Subheading] OR "epidemiology"[All Fields] OR "incidence"[All Fields] OR "incidence"[MeSH Terms] OR "incidences"[All Fields] OR "incident"[All Fields] OR "incidents"[All Fields]

**renal cell carcinoma:** "carcinoma, renal cell"[MeSH Terms] OR ("carcinoma"[All Fields] AND "renal"[All Fields] AND "cell"[All Fields]) OR "renal cell carcinoma"[All Fields] OR ("renal"[All Fields] AND "cell"[All Fields] AND "carcinoma"[All Fields])

**Search strategies for EMbase**

#1  transplantation incidence of renal cell carcinoma {transplantation,transplantation surgery,transplantation organ,surgery transplantation,incidence, incidences, incidence of cases, renal cell carcinoma, hypernephroma, grawitz tumor, grawitz tumour}

**Search strategies for Cochrane library**

#1.  transplantation incidence renal cell carcinoma in Title Abstract Keyword

#2. MeSH descriptor
